# Distinct Adverse Clinical Outcomes of Small and Large Cribriform Patterns on Gleason 7 Prostate Cancer: A Preliminary Study

**DOI:** 10.5152/tud.2023.23076

**Published:** 2023-09-01

**Authors:** Ozgur Kazan, Nilufer Kadioglu, Halil Ibrahim Ivelik, Mehmet Sevim, Okan Alkis, Seref Coser, Ibrahim Guven Kartal, Bekir Aras

**Affiliations:** 1 Department of Urology, Kutahya Health Sciences University, Evliya Celebi Research and Training Hospital, Kutahya, Turkey; 2 Department of Pathology, Kutahya Health Sciences University, Evliya Celebi Research and Training Hospital, Kutahya, Turkey

**Keywords:** Cribriform pattern, Gleason score, ISUP, prostate cancer, recurrence

## Abstract

**Objective:**

We aimed to evaluate the effect of large and small cribriform morphology on survival following radical prostatectomy.

**Methods:**

We included 30 patients who underwent radical prostatectomy with curative intent between 2015 and 2022. Patients with the final pathology of Gleason 7 were included. Patients’ radical prostatectomy specimens were reviewed by an experienced genitourinary pathologist. The diverse growth patterns of Gleason grade 4 were specified as poorly formed/fused glands, cribriform glands, and glomeruloid glands. The cribriform morphology was subdivided into small and large cribriform. Large cribriform growth morphology was defined by its size, which was double that of benign prostate glands. Small and large cribriform glands’ percentages were indicated semiquantitatively. The cribriform morphology subtype present at 50% and higher was defined as the dominant pattern. The effect of histopathological patterns on biochemical recurrence and clinical progression was analyzed.

**Results:**

Thirteen patients were small cribriform pattern dominant (group 1), whereas 14 of the patients were large cribriform pattern dominant (group 2). Pathological T, N stages, and surgical margin positivity were similar between groups. Biochemical recurrence and clinical progression rates were significantly higher in group 2. The large cribriform dominant patients had worse 2-year biochemical recurrence-free survival than small cribriform dominant patients (45.5% vs. 66.7%). In the univariate analysis, International Society of Urological Pathology grade, Gleason pattern 4 percentage, large cribriform pattern dominancy, and pT stage were predictors for biochemical recurrence-free survival. International Society of Urological Pathology grade was the only independent predictor for biochemical recurrence-free survival.

**Conclusion:**

Large cribriform pattern dominancy is associated with worse biochemical recurrence-free survival in Gleason 7 prostate cancer.

**Main Points**

- The study investigated the impact of small and large cribriform morphology on survival in patients with Gleason 7 prostate cancer who underwent radical prostatectomy.
- Patients with large cribriform pattern dominance had worse 2-year biochemical recurrence-free survival (BCRFS) compared to those with small cribriform pattern dominance.
- Large cribriform pattern dominance was found to be a predictor for worse BCRFS, along with other factors such as ISUP grade, Gleason pattern 4 percentage, and pT stage.

## Introduction

Prostate cancer histopathological grading is generated through Gleason grading based on the structural growth pattern of the tumor. Gleason pattern 4 has different growth patterns, such as poorly formed, fused, glomeruloid, and cribriform, according to the 2014 International Society of Urological Pathology (ISUP) classification. International Society of Urological Pathology defined cribriform pattern as “solid proliferation with multiple punched-out lumina.”^1,2^ Gleason pattern 4 differs from other growth patterns with its aggressive behavior. Patients who have cribriform pattern were associated with worse prognosis.^3–5^ The 2005 ISUP consensus had grouped the cribriform morphology primarily on the basis of size and regularity of structure. Smoothly bordered small cribriform nodules were graded as Gleason pattern 3. Large cribriform glands and/or cribriform glands exhibiting irregular borders were graded as Gleason pattern 4. Numerous controversies of small and large cribriform patterns arose between pathologists regarding which group should be counted as Gleason pattern 3 and which as Gleason pattern 4. Most pathologists also considered small cribriform foci as Gleason pattern 4.^6^

All cribriform cancer foci, irrespective of shape and size, are now considered Gleason pattern 4 to increase compliance among pathologists.^7^ Foci with all cribriform morphology were also considered to have an aggressive course in this case. However, the number of studies comparing the clinical prognosis of small and large cribriform patterns is very limited. In one of these studies, the large cribriform pattern was shown to have a worse prognosis than the small cribriform pattern, while the other showed that both the large and small cribriform patterns had a similar aggressive course.^8,9^

The objective of this study was to conduct a comparison of the survival difference between small and large cribriform patterns in radical prostatectomy patients with adenocarcinoma Gleason pattern 7.

## Material and Methods

### Patient Selection

A total of 267 patients who underwent radical prostatectomy with curative intent between January 2015 and June 2022 were screened. Our study included patients who had a final pathology of Gleason 7. Patients with clinical lymph node positivity or distant metastasis, and patients who had undergone hormone therapy before prostatectomy were excluded.

Patients’ demographics and clinical outcomes were retrospectively reviewed. The primary endpoints were biochemical recurrence (BCR) and clinical progression (CP). We defined BCR as a PSA level equal to or greater than 0.2 ng/mL following radical prostatectomy. Clinical progression was determined based on the Response Evaluation Criteria in Solid Tumors (RECIST) applied to bone scans and abdominal computed tomography (CT) scans in response to PSA elevation or symptoms. More recently, CP has also been assessed based on the presence of lesions observed on prostate-specific membrane antigen (PSMA)-PET/CT imaging.

We obtained informed consent from every patient enrolled in the study, and the study itself received approval from the Kutahya Health Sciences University ethics committee (2022/07-15).

### Histopathology

**Figure 1.**
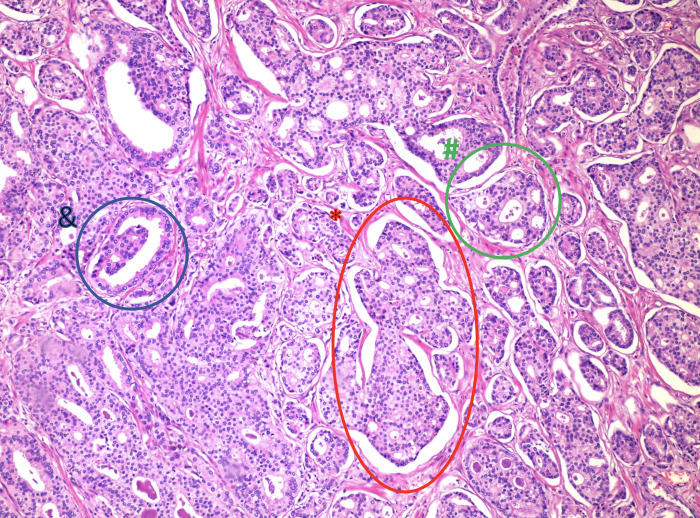
Small and large cribriform glands at the center and poorly formed/fused glands at the periphery on radical prostatectomy specimen. HEX100. ^*^Large cribriform morphology, ^#^Small cribriform morphology, ^&^Glomeruloid morphology.

Radical prostatectomy specimens were re-evaluated by an experienced pathologist blinded to clinical information. The 8th tumor, lymph node, metastasis (TNM) classification and 2014 ISUP classification were used.^7,10^ We evaluated the percentage of Gleason pattern 4. The radical prostatectomy specimens were examined to identify the presence of the cribriform pattern as well as other growth patterns. Gleason pattern 4 tumors were specified as poorly formed/fused glands, cribriform glands without necrosis, and glomeruloid glands; their presence was noted and then their percentages were measured semiquantitatively. Cribriform glands were subdivided as small and large according to their size. Large cribriform glands were defined as at least 2-fold the size than adjacent benign gland size (Figure 1). Small and large cribriform glands’ percentages were indicated semiquantitatively. In the samples, there were small and large cribriform morphologies and also a mixture of both patterns present. Therefore, the percentages of small and large cribriform patterns in each specimen were calculated semiquantitatively. The dominant morphology at 50% and higher was determined as the dominant pattern.

### Statistical Analysis

Descriptive statistical methods (mean, standard deviation, median) were used in evaluating the data. The normality was tested with the Kolmogorov–Smirnov test. To compare qualitative data, statistical tests such as the Pearson chi-square test and Fisher’s exact test were employed. For the analysis of quantitative data, an independent samples *t*-test was conducted. Survival analysis was performed using Kaplan–Meier survival analysis, and the Log-rank test was employed to assess survival differences. The impact of other risk factors on mortality was evaluated through Cox regression analysis. The significance level was set at *P* < .05.

Comparing the BCR rates between large and small cribriform dominant groups, we calculated the effect size of *W *= 0.555 using Statistical Package for the Social Sciences version 25.0 (IBM SPSS Corp.; Armonk, NY, USA). A post hoc test was conducted using G*Power version 3.1 for power analysis. With a significance criterion *α* = 0.05 and 13 patients in each group, the power of our study was calculated at 0.81.

## Results

A total of 30 patients were included in the study. Cribriform morphology was present in 27 (90%) of the patients. In 3 patients, cribriform morphology was not present and those were presented as poorly formed growth patterns. Purely cribriform pattern was dominant in 10 patients. In 17 patients, more than 1 growth pattern was present. Twenty (66.7%) of the patients were identified as ISUP-2 and 10 (33.3%) as ISUP-3. Intraductal carcinoma was positive in 19 patients. The average duration of follow-up in the study was 16.8 months (Table 1).

There were 13 patients who exhibited small cribriform pattern dominancy (group 1) and 14 patients with large cribriform pattern dominancy (group 2). The mean age and preoperative PSA values were similar between the 2 groups. The percentage of pattern 4 was higher in group 2 (50.4% vs. 33.3%, *P* = .026). pT, pN stages, and surgical margin positivity rates were similar in both groups. Biochemical recurrence and CP rates were significantly higher in the large cribriform pattern dominant group (Table 2).

**Figure 2.**
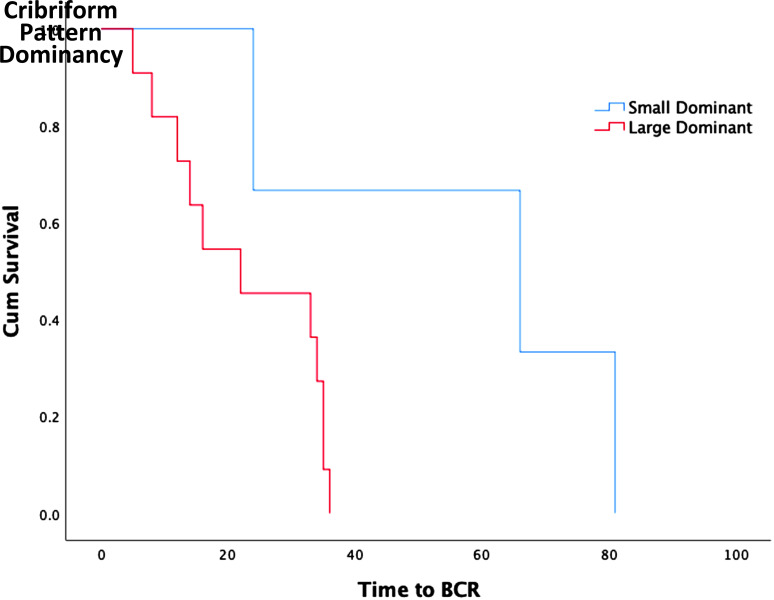
Biochemical recurrence-free survival of small and large cribriform pattern dominant patients.

The 2-year biochemical recurrence-free survival (BCRFS) was statistically significantly worse in the large cribriform pattern dominant group (45.5% vs. 66.7%, *P* = .048) (Figure 2). The 2 groups were not compared, as CP was not observed in the small cribriform pattern dominant group. In the univariate analysis of predictors affecting BCRFS, ISUP grade, pattern 4 percentage, large cribriform pattern dominancy, and pT stage were statistically significant. In the multivariate analysis, only ISUP grade was found to be an independent predictor (Table 3).

## Discussion

In this study, we aimed to analyze whether small and large cribriform patterns differ in terms of survival in Gleason 7 prostate cancer. In this preliminary study with a limited sample size, we showed that patients with predominantly large cribriform morphology had worse BCRFS. We also detected large cribriform pattern dominant morphology as one of the factors predicting BCRFS in the univariate Cox regression analysis, while we found ISUP grade as the only independent predictor of BCRFS.

We defined large cribriform morphology by its size, which is at least twice the size of the benign prostate tissues used in previous studies. Actually, recent studies have defined large cribriform diversely. The definition in our study is the one used most frequently.^8,9^ Trudel et al^11^ defined the large cribriform morphology as the presence of larger cribriform glands than benign glands. Some studies have included the number of cribriform structures and expansion to solid formations without comedonecrosis.^12^ No matter how cribriform morphology is defined, a trend among pathologists has arisen toward reporting any cribriform pattern as Gleason 4.^6^ Iczkowski et al^13^ indicated that any cribriform pattern, large or small, is associated with adverse outcomes regarding biochemical failure. The aggressive nature of the cribriform morphology among pathologists has differentiated this pattern from Gleason grade 3. Russo et al^14^ provided the largest systematic review with 31 studies of the adverse outcomes of the cribriform pattern. They determined that cribriform morphology was associated with worse BCRFS and cancer-specific survival.

In our study, we showed that BCR and CP were more common in the large cribriform dominant group. In the regression analysis, the presence of large cribriform pattern dominance was determined as one of the factors affecting BCRFS.

In the study of Iczkowski et al,^13^ the effect of the presence of small and large cribriform patterns on BCR in radical prostatectomy specimens was evaluated, and no difference was found between the two groups. The presence of any cribriform morphology had a negative effect on BCR. Unlike our study, any presence of small or large cribriform growth structures was considered positive. While 55% of the patients had large cribriform structures, both small and large cribriform structures were detected in the remainder and the groups were analyzed accordingly. In our study, 2 groups were compared on the basis of small or large cribriform dominance. Similarly, in our study, both large and small cribriform growth patterns were seen together at a rate of 55.6%. Therefore, instead of defining the presence of any pattern as large or small cribriform, the distribution of growth patterns was calculated semiquantitatively, and if the presence of 50% or more was observed, the large or small cribriform pattern was defined as dominant. There are 2 more recent studies comparing small and large cribriform patterns. Both studies similarly defined any presence of a large cribriform pattern as a large cribriform pattern. Rijstenberg et al^8^ determined similar adverse clinical outcomes of large and small cribriform morphology on prostate cancer biopsies. Hollemans et al^9^ analyzed ISUP-2 radical prostatectomy specimens and showed that the presence of a large cribriform pattern was identified as an independent predictor of BCR.

Only small or only large cribriform morphology groups could be seen in biopsy specimens, but both groups together are observed commonly in radical prostatectomy specimens because the tumor structure is thoroughly examined. For this reason, counting the small and large cribriform growth patterns and even calculating the area will provide a more accurate histopathological evaluation by calculating the dominance of small and large cribriform patterns and defining it accordingly.

Our study is the first study to define the dominance of small and large cribriform patterns. Leo et al^15^ analyzed the “Cribriform Area Index” by automated image analysis and determined it as a prognosticator of BCR following radical prostatectomy. Similarly, we calculated the small and large cribriform growth patterns semiquantitatively. A limitation of our study is the lack of computationally derived area calculation. Our study has other limitations, including its small sample size and its retrospective design. We intended to present this preliminary study because the cribriform pattern is a frequently discussed morphology in the Gleason grade 4 and has a very valuable prognostic effect, especially since all cribriform morphologies were evaluated in the same prognostic group after 2015. Since we have seen this significant effect of the large cribriform pattern on BCRFS, multicenter studies with a larger patient population should be planned.

This study demonstrated that different subgroups within the cribriform morphology may produce different adverse outcomes. Patients diagnosed with Gleason 7 prostate cancer exhibiting a large cribriform morphology are exposed to worse BCRFS following radical prostatectomy.

## Figures and Tables

**Table 1.** Clinicopathological Features of Patients

Age, years (mean ± SD)	68.7 ± 5.49
PSA, ng/mL (mean ± SD)	16.8 ± 27.1
ISUP grade, %	
ISUP-2	20 (66.7%)
ISUP-3	10 (33.3%)
Percentage of pattern 4, % (mean ± SD)	40.7 ± 19.9
Intraductal carcinoma, %	
Absent	11 (36.7%)
Present	19 (63.3%)
Pattern 4 subtype, %	
Non-cribriform	3 (10%)
Cribriform	27 (90%)
Cribriform pattern subtype, %	
Small	7 (25.9%)
Large	5 (18.5%)
Mix	15 (55.6%)
Percentage of small cribriform pattern, % (mean ± SD)	56.9% ± 38.2%
Percentage of large cribriform pattern, % (mean ± SD)	60.7% ± 35.9%
Large cribriform pattern dominancy	
Absent	13 (48.1%)
Present	14 (51.9%)
pT Stage	
pT2	18 (60%)
pT3a	9 (30%)
pT3b	3 (10%)
pN Stage	
Nx	12 (40%)
N0	16 (53.3%)
N1	2 (6.7%)
Surgical margin	
Negative	21 (70%)
Positive	9 (30%)
Biochemical recurrence	
Absent	16 (53.3%)
Present	14 (46.7%)
Clinical progression	
Absent	23 (76.7%)
Present	7 (23.3%)
Follow-up, months (mean ± SD)	16.8 ± 11.9

ISUP: International Society of Urological Pathology, SD: Standard deviation.

**Table 2.** Clinical Features and Outcomes According to the Small/Large Cribriform Pattern Dominance

	Small Cribriform Dominant	Large Cribriform Dominant	*P*
	n = 13	n = 14	
Age, years (mean ± SD)	68.5 ± 3.6	69.9 ± 6.6	.532
PSA, ng/mL (mean ± SD)	10.5 ± 7.4	24.5 ± 38.3	.208
ISUP grade, %			
ISUP-2	10 (76.9%)	7 (50%)	.148
ISUP-3	3 (23.1%)	7 (50%)	
Percentage of pattern 4, %. (Mean±SD)	33.4 ± 19.9	50.4 ± 17.1	***.026***
Intraductal carcinoma, %			
Absent	5 (38.5%)	3 (21.4%)	.333
Present	8 (61.5%)	11 (78.6%)	
pT Stage			
pT2	8 (61.5%)	8 (57.1%)	.177
pT3a	5 (38.5%)	3 (21.4%)	
pT3b	0 (0%)	3 (21.4%)	
pN Stage			
Nx	5 (38.5%)	6 (42.9%)	.131
N0	8 (61.5%)	6 (42.9%)	
N1	0 (0%)	2 (14.2%)	
Surgical margin			
Negative	11 (84.6%)	8 (57.1%)	.118
Positive	2 (15.4%)	6 (42.9%)	
Biochemical recurrence			
Absent	10 (76.9%)	3 (21.4%)	***.004***
Present	3 (23.1%)	11 (78.6%)	
Clinical progression			
Absent	13 (100%)	7 (50%)	***.003***
Present	0 (0%)	7 (50%)	

ISUP: International Society of Urological Pathology, SD: Standard deviation.

***p<0.05*** statistically significant

**Table 3.** Factors Affecting Biochemical Recurrence

	Univariate Analysis			Multivariate Analysis		
	Odds Ratio	95% CI	*P*	Odds Ratio	95% CI	*P*
ISUP grade	11.8	1.391-100.8	.024	11.8	1.391-100.8	.024
Percentage of pattern 4	1.05	1.008-1.100	.021			
Large cribriform dominancy	6.18	0.769-49.667	.087			
pT stage	2.19	0.923-5.218	.075			
